# Pharmacokinetic modeling of over-the-counter drug diphenhydramine self-administered in overdoses in Japanese patients admitted to hospital

**DOI:** 10.1186/s40780-021-00215-w

**Published:** 2021-08-02

**Authors:** Koichiro Adachi, Satoru Beppu, Mariko Terashima, Wataru Kobari, Makiko Shimizu, Hiroshi Yamazaki

**Affiliations:** 1 Laboratory of Drug Metabolism and Pharmacokinetics, Showa Pharmaceutical University, 3-3165 Higashi-tamagawa Gakuen, Machida, Tokyo, 194-8543 Japan; 2 Kyoto Medical Center, Fushimi-ku, Kyoto, 612-8555 Japan; 3 Present address, Himeji Medical Center, Himeji, Hyogo 670-8520 Japan

**Keywords:** Drug monitoring data, Liver damage, Pharmacokinetic prediction, Total bilirubin

## Abstract

**Background:**

Although the over-the-counter H_1_ receptor antagonist diphenhydramine is not a common drug of abuse, it was recently recognized as one of the substances causing acute poisoning in patients attempting suicide that led to admissions to our hospital emergency room.

**Case presentation:**

Two patients [women aged 21 and 27 years (cases 1 and 2)] were emergently admitted after intentionally taking overdoses of 900 and 1200 mg diphenhydramine, respectively. The plasma diphenhydramine concentrations in case 1 were 977 and 425 ng/mL at 2.5 and 11.5 h after single oral overdose, and those in case 2 were 1320 and 475 ng/mL at 3 and 18 h after administration, respectively. We set up a simplified physiologically based pharmacokinetic (PBPK) model that was established using the reported pharmacokinetic data for a microdose of diphenhydramine. The two virtual plasma concentrations and the area under the curve (AUC) values extrapolated using the PBPK model were consistent with the observed overdose data. This finding implied linearity of pharmacokinetics over a wide dosage range for diphenhydramine.

**Conclusions:**

The determined plasma concentrations of diphenhydramine of around 1000 ng/mL at ~ 3 h after orally administered overdoses in cases 1 and 2 may not have been high enough to cause hepatic impairment because levels of aspartate aminotransferase and alanine aminotransferase were normal; however, there was an increase in total bilirubin in case 1. Nonetheless, high virtual liver exposures of diphenhydramine were estimated by the current PBPK model. The present results based on drug monitoring data and pharmacokinetic predictions could serve as a useful guide when setting the duration of treatment in cases of diphenhydramine overdose.

## Background

Diphenhydramine is an over-the-counter H_1_ receptor antagonist used to treat allergies and to induce sleep, but it is not a common drug of abuse [1, 2]. Although diphenhydramine is often considered to be relatively nontoxic, dose adjustment may be needed to avoid possible hepatic impairment [1]. There have been reported fatal and nonfatal cases of diphenhydramine overdose in the clinical setting [2]. Moreover, there is the potential for an increasing number of cases of deliberate drug poisoning with excessive use [3, 4]. Although diphenhydramine did not rank in the top 20 substances in a drug poisoning cohort study of 2016 [3], it was ranked second among substances causing acute poisoning resulting in patient admissions to the emergency room of Kyoto Medical Center between January 2018 and March 2021 (Table 1**)**.

**Table 1:** Top 11 substances causing acute poisoning from overdoses that led to admission to the emergency room of Kyoto Medical Center

Substance	Number of poisoning cases (%)
1. Flunitrazepam^a^	12 (7.8)
2. Diphenhydramine	8 (5.2)
3. Etizolam ^a^	7 (4.5)
4. Quetiapine	7 (4.5)
5. Ethanol	6 (3.9)
6. Lorazepam^a^	5 (3.2)
7. Triazolam^a^	5 (3.2)
8. Brotizolam^a^	4 (2.6)
9. Diazepam^a^	4 (2.6)
10. Loxoprofen	4 (2.6)
11. Risperidone^a^	4 (2.6)
Total	154 (100)

We conducted a cohort study of 87 patients who self-administered substances that resulted in acute poisoning and admission to the emergency room of Kyoto Medical Center between January 2018 and March 2021

^a^These seven substances are reported to be commonly ingested in cases of deliberate drug poisoning in Japan [3]

The monitoring of plasma concentrations of diphenhydramine should be considered even in emergency situations. The drug monitoring of steady-state plasma concentrations in individual patients in the clinical setting can, in general, be supported by pharmacokinetic models and simulations. Full physiologically based pharmacokinetic (PBPK) models [5] can predict drug monitoring results in patients [6–8]. We have developed simplified PBPK models [9] and have applied them to cases of edoxaban overdose [10] and to an overdose of duloxetine along with other antipsychotic drugs [11]. The practical use of such PBPK models has been suggested for supporting paramedical staff in emergency clinical practice [10, 11].

## Case presentation

**Fig. 1:**
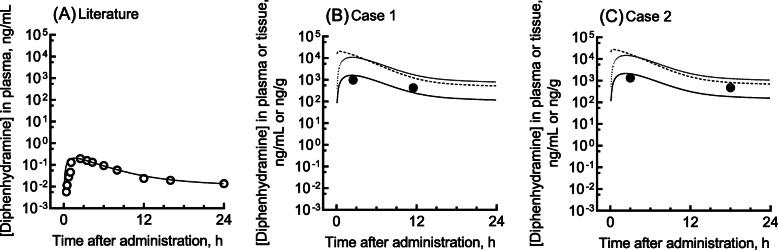
Reported/measured (circles) and estimated (lines) concentrations of diphenhydramine in plasma and/or tissues of healthy subjects (A) and two patients who took single oral overdoses (B,C). Plasma concentrations of diphenhydramine were measured in patients who had taken single oral overdoses of diphenhydramine of 900 mg (case 1, B) and 1200 mg (case 2, C). The modeled plasma (solid lines), hepatic (broken lines), and renal (dotted lines) concentration curves after virtual administrations of 900 and 1200 mg diphenhydramine are also shown. In panel A, reported mean plasma concentrations of diphenhydramine after an oral microdose (0.1 mg) were taken from literature [12]

Here we describe a 21-year-old woman (body weight, 52 kg) and a 27-year-old woman (body weight, 67 kg) (cases 1 and 2) who, as suicide attempts, intentionally took overdoses of 900 and 1200 mg diphenhydramine, respectively (the usual clinical dose is in the range 50–150 mg/day [2]), and were emergently admitted to Kyoto Medical Center, with empty heat seals for diphenhydramine. These patients had no medical history. The clinical laboratory results for these two patients after self-administered diphenhydramine overdoses are shown in Table 2. Figure 1B and C show the two measured plasma concentrations and the PBPK-modeled concentration profiles of diphenhydramine self-administered in single oral overdoses for cases 1 and 2, respectively. The patients gave written informed consent to take part in this study and for its publication. The Ethics Committee of Kyoto Medical Center approved this study (18–018).

**Table 2:** Clinical laboratory results in two patients who had taken single oral overdoses of diphenhydramine

	Time after administration of oral dose			
	Case 1		Case 2	
	2.5 h	11.5 h	3 h	18 h
Aspartate aminotransferase (U/L)	20	26	21	19
Alanine aminotransferase (U/L)	10	11	26	22
Total bilirubin (mg/dL)	1.6	1.5	0.5	0.6
Serum creatinine (mg/dL)	0.67	0.70	0.83	0.79
Creatinine clearance (mL/min)	109	104	108	113

On arrival, case 1 was conscious but was in a state of restlessness and was unable to communicate. Her awareness level was assessed, and the Glasgow Coma Scale score was eye 4, verbal 4, and motor 6 (E4V4M6); she had a breathing rate of 20 breaths/min, a body temperature of 36.9 °C, a blood pressure of 140/80 mmHg, a heart rate of 102 bpm, and a narrow QRS complex on her electro-cardiogram with a QTc of 467 ms. After infusion with bicarbonate Ringer’s solution, the patient’s awareness level 9 h after arrival had improved to E4V5M6 with a breathing rate of 18 breaths/min, a body temperature of 36.8 °C, a blood pressure of 123/80 mmHg, a heart rate of 77 bpm, and a reduced QTc of 449 ms. The patient was discharged 2 days after admission. There was a slight increase in total bilirubin (Table 2), but no other laboratory data abnormalities were noted. In contrast, the awareness level in case 2 was E4V5M6 on arrival. On admission, the patient had a breathing rate of 24 breaths/min, a body temperature of 37.8 °C, a blood pressure of 148/89 mmHg, a heart rate of 128 bpm, and a normal sinus rhythm with a QTc of 476 ms on the electro-cardiogram. However, 15 h after administered, these values had reduced to 18 breaths/min, 36.6 °C, 123/82 mmHg, 85 bpm, and < 430 ms, respectively. No abnormalities were founded in vital signs, and the patient was discharged 4 days after admission. After the patients’ level of consciousness had gradually improved, subsequent questioning revealed that they had taken high doses of diphenhydramine.

Frozen plasma samples collected from the two patients after diphenhydramine overdoses were pharmacokinetically analyzed. The plasma concentrations of diphenhydramine, after being deproteinized with four volumes of acetonitrile, were quantified by liquid chromatography using a gradient elution program followed by tandem mass spectrometry systems, according to previously reported methods [11] with slight modifications. An API4000 tandem mass analyzer (AB Sciex, Framingham, MA, USA) was used in electrospray positive ionization mode and was directly coupled to a Shimadzu LC-20 AD system equipped with an octadecylsilane (C_18_) column (XBridge, 3.5 μm, 2.1 mm × 150 mm, Waters, Milford, MA, USA). The liquid chromatography conditions were as follows: solvent A was 10 mM ammonium acetate buffer (pH 6.8) and solvent B was acetonitrile. The following gradient program was used with a flow rate of 0.20 mL/min: 0–8 min, linear gradient from 35% B to 70% B (v/v); 8.1–10 min, hold at 90% B; and 10.0–15 min, hold at 35% A. The temperature of the column was maintained at 40 °C. Prepared samples (2.0 μL) were injected with an auto-sampler. Diphenhydramine was quantified using the *m/z* 256 → 167 transition in the range of 10–2500 ng/mL. Under the present conditions, diphenhydramine levels in plasma were measurable at concentrations ≥10 ng/mL and were detectable at concentrations ≥0.10 ng/mL. Authentic diphenhydramine was purchased from Fujifilm Wako Pure Chemicals, Osaka, Japan. The measured plasma concentrations of diphenhydramine self-administered in single oral overdoses are shown in Fig. 1. The plasma diphenhydramine concentrations in case 1 were 977 and 425 ng/mL at 2.5 and 11.5 h, respectively, after an oral overdose of 900 mg (Table 3). The plasma concentrations in case 2 at 3 h and 18 h after administration were 1320 ng/mL and 475 ng/mL, respectively, after an oral overdose of 1200 mg diphenhydramine.

**Table 3:** Observed plasma concentrations and PBPK modeled concentrations of diphenhydramine in two patients who had taken overdoses

Pharmacokinetic data	Observed	PBPK output ^a^
Case 1, 900 mg diphenhydramine		
C_2.5_, ng/mL	977	1730 (1.8)
C_11.5_, ng/mL	425	310 (0.73)
AUC_0–11.5_, ng h/mL	7530	10,900 (1.5)
Case 2, 1200 mg diphenhydramine		
C_3_, ng/mL	1320	2110 (1.6)
C_18_, ng/mL	475	220 (0.46)
AUC_0–18_, ng h/mL	15,400	16,400 (1.1)

^a^Values in parentheses are ratios of the calculated values to the observed values after 900 mg (case 1) and 1200 mg (case 2) administrations

We also report the results of pharmacokinetic modeling of plasma and tissue concentrations of diphenhydramine. Based on the reported human blood concentrations of diphenhydramine after subjects were orally treated with a normal or microdose [12, 13] (Fig. 1A), a simplified PBPK model consisting of gut, liver, kidney, central, and peripheral compartments was set up as described previously [10, 11, 14]. The initial values for the fraction absorbed × intestinal availability (*F*_a_·*F*_g_) and hepatic clearance (*CL*_h_) were estimated from the elimination rate constants in empirical one-compartment models. The absorption rate constant (*k*_a_), volume of the systemic circulation (*V*_1_), and hepatic intrinsic clearance (*CL*_h,int_) values with standard deviations (as parameters for the PBPK model) were determined by fitting using nonlinear regression analyses; these final parameters are shown in Table 4. The resulting system of differential equations was solved to obtain the concentrations of the substrates for the overdosed patients in this study:

**Table 4:** Physiological, experimental, and final calculated parameters for the diphenhydramine PBPK model established in this study

Parameter	Value for diphenhydramine
Model input parameters	
Molecular weight	255
Octanol–water partition coefficient	3.45
Plasma unbound fraction	0.216
Blood–plasma concentration ratio	0.898
Liver–plasma concentration ratio	3.27
Fraction absorbed × intestinal availability	0.436
Absorption rate constant, 1/h	1.36 ± 0.01 ^a^
Transfer rate constant (*k*_12_)_,_ 1/h	0.107 ± 0.001 ^a^
Transfer rate constant (*k*_21_)_,_ 1/h	0.0437 ± 0.0001 ^a^
Volume of systemic circulation, L	117 ± 1
Hepatic intrinsic clearance, L/h	100 ± 1
Hepatic clearance, L/h	17.7
Renal clearance, L/h	0.3
Estimated levels	
C_max_ in plasma, ng/mL	0.209 (1.07) ^b^
AUC in plasma, ng h/mL	1.46 (1.07) ^b^
C_max_ in liver, ng/mL	2.93
AUC in liver ng h/mL	10.7
C_max_ in kidney, ng/mL	1.43
AUC in kidney ng h/mL	9.96
Reported values [12, 13]	
Maximum drug concentration time, h [12]	2.5
C_max_ in plasma, ng/mL [12]	0.195
AUC in plasma, ng h/mL [12]	1.36
Half-life, h [12]	12
Bioavailability [12]	0.34
Urinary excretion of unchanged drug [13]	0.01 ^c^

The plasma unbound fraction, octanol–water partition coefficient, blood-to-plasma concentration ratio, and liver-to-plasma concentration ratio of diphenhydramine were estimated using in silico tools [14]

^a^Data are means ± standard deviations by fitting to measured concentrations

^b^Values in parentheses of estimated levels are ratios to the reported values taken from the literature (shown in Fig. 1A**,** [12]) after 0.1 mg administrations

^c^ Urinary excretion ratio was taken from the literature [13] after 100 mg administrations

$\frac{d{X}_g(t)}{dt}=-{k}_a\cdotp {X}_g(t)$ when at *t* = 0, *X*_*g*_(0) = *dose*

$$ {V}_h\frac{d{C}_h}{dt}={Q}_h\cdotp {C}_b-\frac{Q_h\cdotp {C}_h\cdotp {R}_b}{K_{p,h}}+{k}_a\cdotp {X}_g-{CL}_{h,\mathit{\operatorname{int}}}\cdotp \frac{C_h}{K_{p,h}}\cdotp {f}_{u,p} $$

$$ {V}_1\frac{d{C}_b}{dt}=-\left({Q}_h+{Q}_r\right)\cdotp {C}_b+\frac{Q_h\cdotp {C}_h\cdotp {R}_b}{K_{p,h}}-{k}_{12}\cdotp {V}_1\cdotp {C}_b+{k}_{21}\cdotp {X}_{peripheral}+\frac{Q_r\cdotp {C}_r\cdotp {R}_b}{K_{p,r}} $$

$$ {V}_r\frac{d{C}_r}{dt}={Q}_r\cdotp {C}_b-\frac{Q_r\cdotp {C}_r\cdotp {R}_b}{K_{p,r}}-{CL}_r\cdotp \frac{C_r}{K_{p,r}}\cdotp {f}_{u,p} $$

$$ \frac{d{X}_{peripheral}}{dt}={k}_{12}\cdotp {V}_1\cdotp {C}_b-{k}_{21}\cdotp {X}_{peripheral} $$

where *X*_g_ and *X*_peripheral_ are the substrate amounts in the gut and peripheral compartments, and *C*_h_, *C*_r_, and *C*_b_ are the hepatic, renal, and blood substrate concentrations. *V*_h_ and *V*_r_ are the liver (1.5 L) and kidney (0.28 L) volumes and *Q*_h_/*Q*_r_ are the blood flow rates of the systemic circulation to the hepatic/renal compartments (96.6 L/h).

## Discussion and conclusions

Although diphenhydramine did not rank in the top 20 substances involved in overdoses in Japan, we experienced multiple diphenhydramine overdose cases at our hospital. Reportedly, blood diphenhydramine concentration levels of 15–112 ng/mL are considered therapeutic, 1000–5000 ng/mL are considered toxic, and 5000–39,000 ng/mL are considered lethal [1, 2, 4]. In the present study, the two modeled plasma concentrations and AUC values by trapezoidal methods estimated using the PBPK model were consistent with the observed values for single oral overdoses of 900 and 1200 mg diphenhydramine. The relatively good fit of the PBPK-modeled plasma concentrations and AUC values (within a two-fold range of observed values), as shown in Table 3, was noted. Diphenhydramine’s time to reach maximum concentration is reportedly 1.7 ± 1.0 h, with a terminal elimination half-life of 9.2 ± 2.5 h [1].

The determined plasma concentration levels of diphenhydramine of around 1000 ng/mL for the overdoses seen in cases 1 and 2 at ~ 3 h after oral administration would appear not to be high enough to cause hepatic impairment, as judged by the normal levels of aspartate aminotransferase and alanine aminotransferase in our two patients; however, an increase in total bilirubin was seen in case 1 (Table 2). Nonetheless, high virtual exposure of diphenhydramine in livers was predicted by the current PBPK model (Fig. 1B and C). Interestingly, we experienced an unrelated outpatient case with an increased aspartate aminotransferase level of 45 U/L after 150 mg of diphenhydramine; however, drug monitoring data was not available in this patient.

The elimination half-lives of 7.5 h and 10 h calculated from the two available data points for our two cases after overdoses of 900 and 1200 mg were similar to the reported normal values of 9.2 ± 2.5 h [1]. This finding implies linearity over a wide range of doses for diphenhydramine pharmacokinetics, as exemplified by the present two cases. Gastric lavage should not be considered unless a patient has ingested a potentially life-threatening amount of a poison and the procedure can be undertaken within 60 min of ingestion [15]. Even if more than 1 h has passed after administration, in general, gastric lavage and the administration of activated charcoal [16] may be effective in clinical practice for diphenhydramine overdose patients. In hospitals, a simplified PBPK model-based simulator may be of use in reducing the need to routinely measure the blood levels of drugs. The present findings, based on drug monitoring data and pharmacokinetic modeling predictions, could serve as a useful guide for determining the treatment period in cases of overdoses.

## Data Availability

All data generated or analyzed during this study are included in this published article and are also available from the corresponding author on reasonable request.

## Abbreviations

PBPK: Physiologically based pharmacokinetic
AUC: Area under the curve
